# Detection Methods and Clinical Applications of Circulating Tumor Cells in Breast Cancer

**DOI:** 10.3389/fonc.2021.652253

**Published:** 2021-06-02

**Authors:** Hongyi Zhang, Xiaoyan Lin, Yuan Huang, Minghong Wang, Chunmei Cen, Shasha Tang, Marcia R. Dique, Lu Cai, Manuel A. Luis, Jillian Smollar, Yuan Wan, Fengfeng Cai

**Affiliations:** ^1^ Department of Breast Surgery, Yangpu Hospital, Tongji University School of Medicine, Shanghai, China; ^2^ Cellomics International Limited, Hong Kong, China; ^3^ Shanghai Public Health Clinical Center, Fudan University, Shanghai, China; ^4^ Department of General Surgery, Zhongshan Hospital, Fudan University, Shanghai, China; ^5^ Department of Biomedical Engineering, The City College of New York, New York, NY, United States

**Keywords:** CTCs, breast cancer, clinical application, liquid biopsy, detection methods

## Abstract

Circulating Tumor Cells (CTCs) are cancer cells that split away from the primary tumor and appear in the circulatory system as singular units or clusters, which was first reported by Dr. Thomas Ashworth in 1869. CTCs migrate and implantation occurs at a new site, in a process commonly known as tumor metastasis. In the case of breast cancer, the tumor cells often migrate into locations such as the lungs, brain, and bones, even during the early stages, and this is a notable characteristic of breast cancer. Survival rates have increased significantly over the past few decades because of progress made in radiology and tissue biopsy, making early detection and diagnosis of breast cancer possible. However, liquid biopsy, particularly that involving the collection of CTCs, is a non-invasive method to detect tumor cells in the circulatory system, which can be easily isolated from human plasma, serum, and other body fluids. Compared to traditional tissue biopsies, fluid sample collection has the advantages of being readily available and more acceptable to the patient. It can also detect tumor cells in blood earlier and in smaller numbers, possibly allowing for diagnosis prior to any tumor detection using imaging methods. Because of the scarcity of CTCs circulating in blood vessels (only a few CTCs among billions of erythrocytes and leukocytes), thorough but accurate detection methods are particularly important for further clinical applications.

## Introduction

Breast cancer has overtaken lung cancer in 2020 to become the most common type of cancer in the world. The metastatic nature of breast cancer is also of great concern, as metastasis accounts for 90% of malignancy deaths, because of distal progressions of primary tumors (1). One hypothesis is that tumor metastasis is triggered *via* CTCs (circulating tumor cells) that are found in patients’ blood (2). In a successful dispersal process, cancer cells of breast tumors in primary site infiltrate surrounding tissues, enter blood and lymphatic vessels, followed by translocation to distant sites. The ability to escape from blood vessels and adapt to novel microenvironments in the body allow cancer cells to successfully implant, colonize and proliferate in a new site.

Compared to primary cancer cells, circulating tumor cells show a more aggressive ability to mutate, accumulating genetic changes similar to or larger than those of the original tumor through somatic cell mutations and acquisition of additional characteristics within circulation (3). As the dissemination of tumors appears to be mostly *via* the blood, circulating tumor cells that have penetrated the blood vessels to infiltrate potential metastatic sites are of obvious interest (4); for example, many studies have concluded that the blood-borne dissemination is contributed by EMT (epithelial-mesenchymal transition) in patients with breast cancer (5). Recently, many investigations into liquid biopsy have been conducted; a considerable number showed the potential of CTC detection as an effective method for the evaluation of chemotherapy efficacy, early diagnoses and malignancy recurrence, and choice of drug sensitivity (6). Liquid biopsies of CTCs have the following advantages over other tumor markers in the blood: (1) easy to collect, (2) persistent assessment, (3) analysis of the overall tumors burden rather than a limited part of tumors. Given the many benefits of CTCs have in relation to the early diagnosis and treatment of breast tumors, early stage detection of CTCs in blood appears to be the most critical. However, because of the extremely low concentration of CTCs in human blood, detection has always been a technical challenge. This has led to more research and development in relation to detection methods. Recently, the detection and clinical application of CTCs have been gradually incorporated into the field of nanotechnology and genetics. The detection methods of CTCs are changing dramatically, and the clinical applications are becoming increasingly extensive (7). As a result of the increased attention on this critical subject, multiple detection methods have been developed, and these methods can be used to detect circulating tumor cells *in vivo* as well as *in vitro*.

This article sets out the current state of recent developments relating to detection methods, commercial systems, and clinical applications of CTCs, as well as the limitations and prospects of each method for reference during clinical processes and as guidance for researchers and clinicians.

## Detection Methods of CTCs

CTCs are infrequent neoplastic cells, and a high-level detection platform, appropriate equipment and techniques are needed to detect its rather low concentration in blood, as approximately one CTC per milliliter of blood can escape from primary neoplasms or metastases. The properties to detect and separate heterogeneous CTCs of all types, as well as to separate out the large blood and white cells in blood, are necessary to for a CTC testing platform. With results of negative or positive enrichment, and recognizing circulating tumor cells or removing hematopoietic cells through immune affinity strategy, the selection of CTCs is commonly the first stage of detection. The detection procedure then further distinguishes (and possibly characterizes) CTC from other regular cells. Then, CTCs are identified and isolated from other normal blood cells (8).

### *In Vitro* Detection Methods

CTCs counts have long been seen as a preferred tool in clinical diagnosis. Recently, accurate detection of CTCs may soon be achieved due to many advanced techniques being developed. Enrichment as well as detection procedures are basics for these novel techniques. The gathering and detection methods of CTCs are based on biological or physical features. The latter depends on size, electrical properties, and other physical characteristics of tumor cells, while the former depends on antigen-antibody combinations and the differences of DNA and RNA which distinguish CTCs from normal cells.

#### Detection Methods Relying on Physical Properties

CTCs can be identified from normal cells in blood, such as leukocytes, through physical isolation, without any biomarker labeling. The isolation tactics focus on several distinctions in physical characteristics between cells in blood and CTCs, as set out in **Table 1**.

1. *ISET (Isolation by Size of Epithelial Tumor cells):* Because of their larger size compared to leukocytes in blood, CTCs can be easily isolated by diameter. An identical hypothesis, the nucleoporeassay, is also based on size differences (9, 10). Overall, ISET equipment allows the isolation of CTM (circulating tumor microemboli) and CTCs from all kinds of malignancies as intact cells, without any previous selection based on immune system. However, proof of the existence of CTCs smaller than 8 μm has not yet been explored, leaving questions about the sensitivities of the method.
2. *ScreenCell system:* Through utilizing a microporous membrane filter, ScreenCell systems separate CTCs by cell size screen, making later characterization and sorting possible. Filtration membranes, based on microfiltration technology, enable nucleated cells to pass while holding up CTCs. The potential to achieve high throughput persistent processing of large volumes of blood has been demonstrated. This method has some advantages, for example, related cellular and molecular biological techniques, and techniques that are related to the figures and identification of CTCs and their latent gene abnormalities, can conveniently analyze neoplasm cells extracted onto the filter. Furthermore, cells isolated from blood can undergo tissue culture to be used in future experiments, without the usage of any assay based on antibody, which can open up a broad range of isolation areas for cancer cells, covering non-epithelial originated cells.
3. *Electrical features (Surface charge):*

**Table 1:** The strategies for isolation based on physical properties of CTCs.

Physical characteristics	
Base on Density	Ficoll-Paque^® ^(Density Medium Centrifugation)
Charge on Surface	depFFF (Dielectrophoretic field-flow fractionation)
	ApoStream^®^
Both Size and Density	Onco-Quick^®^ (Porous barrier for size-based separation and density-based centrifugation)
Both Size and Deformability	Vortex chip
	Microfluid vortex capture
Size Only	DFF (Dean Flow Fractionation)
	SSA (Selective size amplification) in conjunction with MOA (multi obstacle architecture)
	ISET^®^ (Isolation by Size of Epithelial tumor cells)
	ScreenCell (Microporous membrane filter)

The majority of cells of mammals have a net negatively-charged surface, especially in physiological situation. However, because of the existence of some macromolecules that have polarized particles, some cell surfaces have complex dielectric features, such as proteins, nucleic acids and polypeptides.

For example, compared to leukocytes in blood, neoplasm cells (e.g. CTCs) have a surface with more zeta potential or negative charge, which has been demonstrated by studies. In addition, these cells, compared to white blood cells, were also proven to have lower cytoplasmic conductivity as well as higher unit membrane capacitance (11).

#### Detection Methods Based on Immunological Properties

This method identifies CTCs by proteins on the cell surface or DNA/RNA in cancer cells. Antibodies in blood can identify and combine with specific markers, usually proteins, on cell surfaces. For instance, epithelial markers are commonly used to identify and separate blood cells and malignancy cells, due to features commonly expressed on epithelial and epithelial cancer cells, which are rarely expressed on the mesenchymal white blood cells.

Immunology-based technology commonly uses specific protein biomarkers, expressed by either cancer cells or blood cells only, and their complementary antibodies. Circulating tumor cells isolation tactics based on proteins can be categorized into 3 types, as set out in **Table 2**.

**Table 2:** The strategies for isolation relied on biological features of CTCs.

Biological characteristics	
Positive Selection	EPHESIA CTC-Chip
	CellSearch^®^ system
	MagSweeper™
	CTC-Chip and Herringbone-Chip
	IsoFlux
	Adnatest^®^ system
	Velcro-like device
	GEDI microdevice
	DEPArray^®^
Negative Selection	leukocytes depletion kits
	RosetteSep method
	CTC-iChip
Selection-free	Epic Sciences
	AccuCyte^®^ – CyteFinder^®^

1. Positive selection: Specific markers usually expressed on the surface of epithelial cancer cells or normal cells, such as epithelial markers, are combined with antibodies, a design to identify CTCs in samples of liquid biopsy. Usually, two steps are involved in these technologies, firstly CTC-enrichment, followed by the step of enumeration and CTC detection. Current positive selection detection procedures include the CellSearch^®^ system, Adnatest^®^ system, MagSweeper™, CTC Chips and Herringbone Chips, EPHESIA CTC Chips, IsoFlux, Velcro-like devices, GEDI microdevices, and DEPArray^®^.

For instance, the US FDA (Food and Drug Administration) has approved the CellSearch^®^ test system, which combines immune-driven nano-magnetic beads with epithelial cell adhesion molecules (EpCAM) through fluorescent specific CTC reaction recognition. It can be applied to breast carcinoma and other malignancies treatment in addition to patient monitoring after chemotherapy or surgeries. However, independent validations of this novel technology are lacking.

2. *Negative selection*: Opposite to the former technology, based on surface markers of cells that are quite different to non-CTCs, consumption procedures are used by negative selection technology to remove white blood cells (WBCs) and other blood cells. In negative CTC enrichment, WBCs are depleted using antibodies of specificity against various biomarkers, such as CD66b or CD45; this makes the selection of leukocytes appearing in blood possible. However, not all nucleated cells in blood are positive for CD66b or CD45. Negative selection methods include white blood cells depletion kits, CTC Chips, RosetteSep method, and Easysep method.

For instance, the Easysep method is quick, simple, and does not require a separation column of immune cells using magnetic separation technology, along with other advantages. This method depends on the TAC technology: normal cells are labeled with antigen-antibody complex, under the action of immune magnetic beads, and normal cells were placed in the EasySep™ magnetic pole, while CTCs were allowed to be negatively selected and can thereafter be extracted from peripheral blood mononuclear cells (PBMCs).

3. *Selection-free* CTCs can express EpCAM at varying levels (12). Selection-free techniques for CTC recognition have been developed, in that some cells express both mesenchymal and epithelial phenotypes. This method combines high throughput microscopy, flow cytometry and RT-PCR. No extra loss of CTCs during a selection procedure is one of the advantages of these methods. Nevertheless, these methods also have some restrictions, for example, CTCs and WBCs need to be distinguished by imperfect biological markers. Examples of selection-free methods include Epic Sciences and AccuCyte^®^ – CyteFinder^®^.

### *In Vivo* Detection Methods

#### Optical Imaging Technology

Clinically, commonly used imaging technologies include MRI (magnetic resonance imaging), PAT (Photoacoustic tomography), OCT (optical coherence tomography), CT (computed tomography) and PET (positron emission tomography). Optical imaging technologies, such as PAT and OCT, are much higher in velocity and resolution than those of CT, MRI, and PET, and can even identify circulating tumor cells *in vivo*. Optical imaging has been proposed for real-time imaging of rapidly flowing cells in blood vessels, including cancer cells (13). Zerda et al., successfully detected free RPMI-8226 myeloma cells in the blood vessels of rat ears by means of speckle-modulating OCT (SM-OCT), which can modulate speckle noise (13) and gold nanorods (~100 nm× 30 nm) were exploited as contrast agents to label cancer cells. OCT technology was first used for single cell detection in the circulatory system within animals. The results show that optical imaging techniques with high resolution can be taken to capture fluorescence-labeled cancer cells and non-labeled melanoma cells or intravascular nanoparticles. However, precisely quantifying the dynamic change of CTCs in fast flowing blood is still very difficult because of the slow imaging velocity of these technologies.

In *in vitro* flow cytometry (IVFC), as each cell passes through the laser beam, fluorescent markers on cell surfaces can be suddenly activated to emit a fluorescence. Quantification of the fluorescent-labeled cells is done through data processing and analysis. The natural blood circulation is directly exploited by IVFC as the fluid flow system to quantitatively analyze the changes of fast flowing target cells in real-time. Different signals are produced by target cells (CTCs) and other cells using different methods such as contrast, fluorescence, photothermal, photoacoustic, light scattering intensity, etc. The methodology for each type of flow cytometry is based on those signals, such as *in vivo* fluorescence flow cytometry, *in vivo* photoacoustic flow cytometry. Fluorescence IVFC (IVFFC) and photoacoustic IVFC (IVPAFC) are the most widely used for CTC detection.

##### Fluorescence Flow Cytometry

A novel small animal research tool was developed by Tan et al. called “Diffuse *in vivo* Flow Cytometry” (DiFC), which is used to directly detect CTCs in blood, that are fluorescently-labeled. Near-infrared diffuse photons are used in this technique, making it possible to count and identify cells flowing in veins and large superficial arteries, and drawing of blood samples is not necessary. The speed and depth can be measured and individual cells of different directions in arterial or venous can be counted when DiFC is combined with a new signal processing algorithm. The experiment shows that all the blood of a mouse can be easily monitored in under 10 minutes, while a false positive rate of 0.014 per minute is maintained by DiFC, which means DiFC is a credible tool to detect CTCs at concentration levels even below 1 cell per milliliter. Therefore, DiFC is a promising technology to be applied in biological applications. In summary, IVFFC has been widely used to monitor the dynamic changes of fluorescently labeled CTCs in the blood circulation system of animal tumor models, including single CTCs, CTC clusters and apoptotic CTCs (14).

##### Photoacoustic Flow Cytometry

IVPAFC was first developed by Zharov et al. of Arkansas University. The mechanism of action is as follows: IVPAFC irradiates blood vessels with a high-frequency pulse laser and detects the instantaneous ultrasonic signals generated by target cells flowing through the blood vessel through ultrasonic detectors placed on the surface of tissue. The target cells absorb light energy and then convert the light energy into heat energy, when the tissue is irradiated. This causes the local temperature to rise, which initiates a thermoelastic change in the tissues, producing an ultrasonic wave (i.e., photoacoustic signal). Compared to detection methods of CTCs *in vitro*, IVPAFC has distinct merits. For instance, in the case of CTCs metastases along lymphatic vessels, lymphatic system and vessels should be enhanced and displayed by contrast agents because of the small structure of lymphatic vessels, as well as the transparent lymph liquid in the lymph vessels.

Besides, the collection amount of lymph fluid is often too small with long-term intubation, causing multiple restrictions in the analysis of CTCs in lymph-based *in vitro* detection methods. In CTC detection by IVPAFC, the background noise in photoacoustic signals produced by colorless lymph is low, which has significant advantages. In the study, the researchers used gold-plated carbon nanotubes and ligand-functionalized iron trioxide magnetic nanoparticles to specifically mark breast tumor cells MDA-MB-231 to evaluate detection levels of target cells. Based on the same technique, others evaluated the detection of CD44 expressing breast cancer stem cells. They used IVPAFC to detect whether CTCs in breast cancer patients have transferred to bone tissue and cerebrospinal fluid, and eventually proved that not only can IVPAFC detect melanoma CTCs *in vivo* in real-time, it can also eradicate melanoma cells and have significant therapeutic effects.

#### Scaffold Implants

The implanted *in vivo* scaffold-based capturing technology mainly stimulates the microenvironment *in vivo*, through biomaterial scaffolds, to induce the migration of cancer cells into the scaffolds. Shea et al. (15) implanted a Polylactide-co-Glycolide (PLG) scaffold into mice and the results showed that PLG scaffolds have the abilities to regulate the local immune microenvironment, capture and recruit metastatic cancer cells, and even decrease solid organs’ tumor burden. All the biomaterials that used have great biocompatibility and can be made into tissue engineering scaffolds or drug sustained-release carriers, etc., which have broad biomedical applications. Furthermore, other types of scaffolds could be used as targets for SUM1315 breast tumor cells to metastasize in mice, such as BMSCs (human bone stromal cells) and BMP-2 (bone morphogenetic protein-2). For such patients, particularly those with a high risk of recurrence, Scaffolds implantation can not only help find tumor metastasis at a relatively early stage, but also treat breast cancer with a relatively low burden.

#### Intravenous Indwelling Needle Device

Intravenous indwelling needles (IINs) mainly consist of a puncture needle core and an endovascular catheter. The surface of the needle core tip is functionalized, allowing high affinity ligands such as antibody and aptamer to bind specifically to CTCs. Another method is when the needle core is replaced with other biologically compatible materials, whereby CTCs can be captured by inserting a catheter into blood vessels.

Presently, GILUPI CellCollector^®^ is the first and only technology approved by US FDA that captures CTCs through IINs. The functionalized thread was laxly pushed into the elbow venous vessel through the indwelling needle’s catheter (blood flow was about 20 mL per min) and stayed about 30 minutes in order to capture CTCs. Thereafter, the thread was taken out, washed, fixed, sealed and immunostained it, and observed and CTCs were counted under a microscope. CTCs are recognised when cells show positive EpCAM or cytokeratin (CK) 4, 5, 8, 9, 18 and are between 10 and 50 micrometers in size and have a high cytoplasmic ratio. The benefit of this method is that it is straightforward and quick, brings little trauma and is easily accepted by patients with breast cancer.

#### Microfluidic Chip

Based on a sorting chip microfluidic cell, through an arteriovenous shunt implanted in mice, the microfluidic chip system has been tested when it is continuously connected to a conscious mouse. When the blood passes through the equipment, microfluidic valves which are pneumatically controlled are able to capture CTCs, and the remaining blood without CTCs will be go back into the mouse. An optofluidic system presented by Hamza (16) showed that the system continuously collected CTCs labeled by fluorescence from a genetically engineered mouse model (GEMM) for several hours per day over various days or weeks. Two-step microfluidic chips were developed by Hyun (17), which can not only sort out heterogeneous CTCs based on their features, but also isolate CTCs from cells in blood. In conclusion, the microfluidic chips are beneficial to research on heterogeneity of CTCs, and by extension, personalized tumor therapy. However, because of the traumatic features of the insertion of catheter in veins and arteries as well as the sterility requirement of CTCs sorting devices and the materials’ biotoxicity, the application of microfluidic chip is limited.

## Clinical Application of CTCs in Breast Cancer and Other Cancers

In 2020, breast cancer is on the top of malignancy lists in the world, especially in developed areas. Identifying/diagnosing cancer and metastatic tumors in an early stage is critically important for clinicians to detect and treat breast cancer patients. Although the prognosis is better than lung cancer and other malignancies, a prominent characteristic of breast cancer is early metastasis, to areas such as lungs, bones and brain, causing the elevated mortality rates of breast cancer. As a prognostic tumor marker of treatment efficacy and metastatic progress with huge potential, CTCs count has been proven and studied by multiple researchers (18). Compared to conventional imaging diagnosis and tissue biopsy, CTC is expected to be a non-invasive tumor screening method for clinical application. In addition, compared to conventional tissue biopsy, liquid biopsy captures the heterogeneous essence of tumors, given the dual sources of primary and metastatic tumors (19,20). Because of that, increasingly more attention has been paid to clinical applications of CTC detection in breast cancer.

### Screening and Early Detection of Cancer

Early diagnosis plays a key role in decreasing mortality of breast cancer, while various grades and types of breast cancer have different significance. For instance, the progress speed of grade I is much more slow than grades II and III of breast cancer, which means early diagnosis when the diameter of tumors is smaller than two centimeters can improve prognosis and OR (overall survival) of patients with breast cancer (21). CTC detection plays a critical part in the screening, diagnosis, early detection and treatment of breast cancer.

A quick CTCs detection method with great sensitivity has been created by Kruspe et al. (22). CTCs‐derived nucleases, a special enzyme that are usually elevated in multiple malignancies, are used for signal amplification in the study. The result shows that probes activated by fluorescent nucleases are able to detect CTCs levels quickly, and can be an economical and effective detection method of CTCs for early diagnosis of breast cancer. Although CTC detection can detect breast cancer earlier than conventional detection methods, further research on CTC detection and its application in tumor diagnosis still needs to be conducted, as using CTC for cancer diagnosis is still in the preliminary phase of development.

### Predicting Recurrence and Prognosis in Breast Cancer

It is commonly accepted that breast cancer malignant tumors have a strong invasive ability that can migrate into remote organs, such as bones and lungs, at an early stage. Therefore, forecasting prognosis and cancer recurrence in breast cancer *via* CTC detection plays a critical role in the treatment. There is a plethora of studies which shows, as an independent predictor, that the number of CTCs in blood before therapies have a significant implication in both OR (overall survival) and progression-free survival of breast cancer patients (23).

The largest multicenter study was published by Rack and his colleagues (24). In this study, they recruited 2,026 early breast cancer patients in group 1 and 1,492 patients in group 2. Group 1 consists of patients before adjuvant chemotherapy, while group 2 consists of patients after chemotherapy, and both groups are treated with the CellSearch System to count the number of CTCs.

According to the study, 21.5% of patients examined were detected with CTCs in their blood, while CTCs can be found in 22.4% of node-positive patients and 19.6% of node-negative patients. 22.1% of patients were detected with CTCs even after chemotherapy. The continuous existence of CTCs in peripheral blood is related to a worse prognosis, in the other words, increased risk of recurrence in primary sites is related to an elevated number of CTCs detected, before or after adjuvant chemotherapy. Another similar study by Madic et al. (25) probed the correlations of CTC and ctDNA plasma level with the prognosis in patients with TNBC (triple negative breast cancer). The study showed that worse prognosis and lower overall survival and time of progression were related to those patients with higher CTC counts. These studies point toward the critical role of CTCs in forecasting relapse and prognosis in patients with breast cancer.

### Recognition of Resistance Mechanisms and Therapeutic Targets

ER/PR and HER-2 are therapeutic targets of breast cancer. Clinical data show that in approximately 30% of cases, the HER-2 status of distant metastatic tumor cells and CTCs are significantly different from that of cells in the primary site, suggesting that drugs targeting the primary tumor may not be appropriate for metastatic tumors. Detecting new properties and characteristics of tumors through conventional tissue biopsy may hurt patients; a non-invasive method like CTCs is an ideal alternative. Other than ER/PR and HER-2, in recent years, the discovery of immune checkpoint regulators such as PD-L1 has made the successful therapy of advanced breast cancer possible, which has become an exciting new therapeutic target. The evidence that PD-L1 is expressed on the surface of CTCs in breast cancer patients with ER or PR(+) and HER2(-) was first found by Mazel and his colleagues (26), and the established assays of CTC/PD-L1 will be used for liquid biopsy in future clinical experiments of breast cancer patients who are experiencing the immune checkpoint.

### Real-Time Monitoring of Therapies

After therapy such as surgery or chemotherapy, real time detection of the level of CTCs in patients is needed to monitor and evaluate their conditions and furthermore, predict and assess survival period. During chemotherapy, the clinical validity of CTCs quantification *via* CellSearch method for monitoring was assessed in metastatic breast cancer patients through analyzing each patients’ real-time data of CTCs (27). Bidard et al. (28) contacted 51 European centres and requested them to offer both reported and unreported anonymized data for individual metastatic breast cancer patients who took part in experiments between January 2003 and July 2012. They concluded that when added to full clinicopathological predictive models, CTCs count can improve metastatic breast cancer’s prognostication, while tumor markers in serum cannot, which means real time monitoring of CTCs can not only detect and identify deterioration, but also be used in the assessment and monitoring of therapies.

### The Clinical Application of CTCs of Other Common Cancers

Through current techniques of CTCs detection, different cancer types can be identified, this is usually done based on the physical, chemical, and biological properties of the tumor cells. For example, the source of the tumor can be determined by detecting CTC phenotypes, immunohistochemistry, and examining the genes of the cells. For example, KRT7 and TTF-1 positive CTCs are associated with lung cancer, PSA and PSMA positive cells are associated with prostate cancer, and KRT20 and CDX2 positive cells are associated with colorectal cancer. Therefore, the tumor type can be identified through the characteristics of circulating tumor cells. In addition to breast cancer, CTC detection technology is also widely used in clinical detection and clinical treatment of a variety of tumors, such as prostate cancer, digestive tract cancer, small cell lung cancer, etc. The CellSearch^®^ system is the only US FDA-approved test platform for CTC isolation, primarily for breast, prostate, and colorectal cancer, and is considered the gold standard for CTC testing. In metastatic prostate cancer, breast cancer, and colorectal cancer, ≥5 circulating tumor cells per 7.5 mL of blood are often associated with poor overall survival and prognosis (23, 29, 30).

Prostate cancer is the most commonly occurring cancer, besides breast cancer. Several large phase II and III trials have established the prognostic value of CTCs in advanced prostate cancer, especially metastatic castrated prostate cancer. The test of CTC has gone beyond traditional PSA measurements to prove to be the strongest independent predictor of survival and has been approved by the FDA for monitoring prostate cancer treatment (30, 31). However, in the hormone-sensitive stage and early stage of prostate cancer, the clinical application of CTC is still a huge challenge due to the very low level of CTCs. In other words, the clinical application of CTC in prostate cancer largely depends on the progression stage of the prostate cancer itself.

Although the CellSearch^®^ system has not been approved for CTCs detection for lung cancer, a large number of clinical trials have proved that CTCs detection has high clinical value in lung cancer, and CTCs technology has been proven to be a predictor of lung cancer prognosis. The number of CTCs in small cell lung cancer is ten times that in non-small cell lung cancer, so circulating tumor cells detection methods are mainly used in small cell lung cancer (32–34). Real-time monitoring of CTCs in the treatment of lung cancer enables prediction of disease progression before clinical deterioration occurs. Similar to breast cancer, a decrease in the number of CTCs in the blood after surgery or chemoradiotherapy often indicates remission of the disease. On the contrary, an increase in the number of CTCs is often indicative of tumor metastasis and recurrence. Interestingly, when He’s team detected CTCs in 165 patients with benign pulmonary nodules, they found 5 CTC-positive patients, and all of these patients developed lung cancer in the next four years, suggesting that CTCs may have a unique role in predicting the occurrence of lung cancer. T J N Hiltermann (35) pointed out that the number of CTCs at baseline and the dynamic change of CTCs after one month chemotherapy is an independent predictor of small cell lung cancer. The number of CTCs has no correlation with tumor response in small cell lung cancer, so when forecasting the survival rate of the patients, compared to the size and volume of tumor, spread of CTCs in blood is more important. In addition, the role of CTCs in the clinical prognosis of cancer has been widely recognized, and it is used as an influencing factor to complement the traditional TNM staging system, and is widely used in the diagnosis, screening, long-term monitoring of lung cancer, as well as to guide the treatment of lung cancer patients.

In non-metastatic urinary carcinoma of the bladder, the detection of CTCs was positively correlated with worse progression-free survival, overall survival, tumor-specific survival and relapse-free survival. In addition, in pure and variant urinary carcinomas of the bladder, CTCs are an independent prognostic factor (36). However, the presence of CTCs in patients with seminogenic and non-seminogenic testicular germ cell tumors is currently unknown (37). Although EpCAM is often negative or very low in renal cell carcinoma, one study claimed that it can detect CTC in 16% of patients with metastatic renal cell carcinoma, and although the detection level is low, it offers a glimmer of hope for the application of CTCs in kidney cancer.

CTC detection technology is also widely used in digestive tract tumors, and CTC count is significantly correlated with the metastasis and recurrence of digestive tract tumors, especially colorectal cancer (36). Guus van Dalum et al. (38) conducted a 5-year follow-up of 183 colorectal cancer patients from Mayo Hospital in the United States, and found that tumor patients with a higher level of CTCs before surgery, had a shorter disease-free survival and poorer prognosis after surgery. Surprisingly, the appearance of CTCS a few weeks after surgery was not significantly correlated with RFS (recurrence-free survival) and CCRD, while the appearance of CTCS two or three years after surgery was significantly correlated with RFS and CCRD. Preoperative CTCs in stage I-III CRC patients were associated with significantly reduced RFS and CCRS. This suggests that the preoperative level of CTCs can guide the chemotherapy of cancer patients. Similarly, in esophageal and gastric cancers, higher preoperative CTCs were associated with poorer postoperative outcomes (39). In pancreatic cancer, the detection rate of CTCs in metastatic pancreatic cancer is significantly higher than that in non-metastatic pancreatic cancer, and the level of CTCs has been shown to be one of the independent influencing factors for overall survival after surgery. However, it is difficult to detect circulating tumor cells from pancreatic cancer in peripheral blood, because CTCs are mostly blocked by the liver in the portal vein. The presence of a large number of CTCs in the portal vein is often indicative of liver metastasis. Therefore, a low level of CTC in peripheral blood and a high level in the portal vein is an important feature of the blood distribution in CTC analysis of pancreatic cancer. A study of 88 patients with cholangiocarcinoma (CCA) by Ju Dong Yang et al. (40) showed that CTCs were detectable in patients with CCA and were an independent predictor of survival in patients with CCA.However, given the small sample size of the study and the absence of continuous blood tests, it is not possible to determine whether CTCs predict overall survival in patients with CCA.

Although CTC applications can be used in a wide range of carcinoma, such as breast, prostate, rectal, small cell lung cancer and pancreatic cancer, CTC detection is not suitable for all tumors (41). According to some studies, the detection of CTCs in a small number of gynecological tumors, neuroendocrine tumors, head and neck tumors, and melanoma has not achieved significant results. This may be related to the mode of metastasis of the tumor itself, the affinity of antigens and antibodies on the tumor surface, and the low number of CTCs in the blood. For example, in recent years, although trace amounts of CTCs have been detected in squamous cell carcinoma of the head and neck, this largely depends on the test method, the timing of blood collection, and the patient’s clinical stage. Therefore, it is difficult to apply CTC detection techniques in the clinical treatment of head and neck tumors. However, if CTCs could be captured under EMT, it would greatly improve the detection of CTCs in head and neck tumors. Therefore, large-scale clinical trials are necessary for head and neck tumors, melanoma and other tumors with very low numbers of CTCs.

## Conclusion and Discussion

Breast cancer is a malignant tumor usually known for its early metastasis and high heterogeneity and cure rates. Early detection and treatment can significantly elevate survival rates in breast cancer patients. CTCs have been proven to play a critically important part in the diagnosis and therapy of breast cancer. There are numerous CTC detection methods, which can be generally divided into *in vivo* and *in vitro* methods, and can be chosen on the basis of the requirements of the specific disease. At present, in order to isolate CTCs with integrity, high vitality and high purity, *in vitro* detection technology is able to capture and release CTCs mildly, construct the negative selection method of specific enrichment and remove normal blood cells to obtain CTCs, and integrate the modules of *in situ* culture and analyze the enriched CTCs immediately. There are two main methods for quantitative analysis of CTCs *in vivo*. One is optic-based IVFCs, which counts CTCs by optical signals. The other is to capture CTCs, then stain tumor cells, and count the number of cells under a microscope. Among them, the commercial CellCollector is the most representative. The currently developed *in vivo* capture technology of CTCs effectively ensures the quality of CTCs before and during capture, but downstream analysis of CTCs after *in vivo* capture is faced with such problems as cell inactivation; new techniques need to be developed to meet standards of CTC culture and molecular analyses. In any case, the detection and molecular characterization of CTCs remains challenging in that they are exceedingly rare and the number of samples available is extremely limited. Therefore, there is an urgent demand to develop a novel system with higher sensitivity, convenience, and efficiency. Clinically, compared with the conventional “gold standard” of tissue biopsy, CTC detection and monitoring can be acquired by routine blood sampling and is an innovative technique using less invasive methods that can sample consistently and repeatedly, so it is safer and timelier.

However, CTCs face many problems in terms of detection technology, clinical application and biological challenges. At present, there are many techniques and methods to detect CTCs in blood, but all of them are faced with the issue of poor sensitivity, which may lead to the delay of detection of tumor cell metastasis in patients, thus delaying treatment. Compared with traditional two-dimensional scaffolds, the implantation of three-dimensional scaffolds and the increase of antibody loading can greatly improve the sensitivity of CTC capture. In addition, although there are many different detection techniques, not one single technique can detect all different types of circulating tumor cells.

Through the detection of CTCs, we not only expect to obtain the dynamic change in the number of CTCs with the development of the tumor, but also hope to obtain biological information related to the phenotype, genome, transcriptome, proteome and metabolome of the primary tumor from CTCs, which are of vital importance in the field of clinical treatment and biological research. Current biological techniques only guarantee the activity of CTCs before and during capture, but *in vitro* downstream analysis of CTCs is faced with problems such as cell inactivation and distortion, which greatly affects the application of CTCs in clinical and biological engineering. Therefore, there is an urgent need to develop a method to isolate CTCs *in vitro* with a mild and specific technique for releasing captured CTCs, so that CTCs can be completely released from the surface of detection system. There is currently a lack of broad consensus on CDC isolation, extraction, collection and preservation of samples, and selection of biomarkers. In order to improve the accuracy of CTCs tests, a standard set of performance assessment criteria should be developed, including enrichment, cell viability and release efficiency, capture efficiency, and purity. This will improve the sensitivity and specificity of CTC detection and maximize the use of CTCs in the blood (which are already scarce).

Although CTCs have great potential in real-time monitoring, prognosis and diagnosis of tumors, CTCs alone cannot be used as an effective indicator to guide the formulation of clinical treatment plans for tumors. Large-scale clinical studies can be carried out in combination with CTCs and other tumor markers, so as to develop the index of circulating tumor cells combined with tumor markers to guide the formulation of clinical tumor programs, which will bring more far-reaching significance and improvements to the applications of circulating tumor cells in clinical and bioengineering. For example, CTC testing in combination with LDH measurements has been shown to be superior to baseline serum LDH measurements alone, in the evaluation of therapeutic efficacy (31). In addition, the biomolecular properties of CTCs have extremely important properties, which can be used to evaluate tumor consistency, prediction of site, specific metastasis, drug resistance map and identification of new drug targets, etc. Therefore, different subsets of CTCs may lead to different clinical outcomes. This gives us an important clue that the analysis of biomolecular properties of individual circulating tumor cells can be used as a novel prognostic or predictive marker to improve patient clinical outcomes. In recent years, CTC research on single biological molecular characteristics has made great progress, but due to the complex detection technology of CTCs, there is still no single enrichment method that can collect each and every CTC in blood, and separate the target cells and unspecific cells completely.

In general, the detection technology of CTCs is improving with each passing day. It is hoped that the current difficulties will be overcome in the near future, and CTC technology will be more widely applied in clinical practice. As an emerging diagnosis and treatment method, CTCs provides the possibility to find and treat breast cancer even at a stage before imaging methods can be used, guide treatment in conjunction with other tumor markers, monitor the treatment of patients after surgery, and predict the prognosis of patients. In addition, there is growing evidence that the current technology can be partially personalized for tumor types, and CTCs can also be applied to other cancers, such as prostate cancer, small cell lung cancer, straight colon cancer, etc., indicating that CTCs will significantly advance the development of precision oncology in the coming decades. However, CTCs still face great clinical and biological challenges, such as low sensitivity, small numbers, loss of cell activity and so on. Although it will be challenging to improve the detection rate of CTCs, make full use of CTCs for gene analysis of the primary tumor, and guide new treatments according to the molecular targets on the surface of CTCs, this also brings great hope for the further development of biology and emerging immunotherapy techniques.

## Author Contributions

HZ, XL, YH, MW, CC, ST, MD, LC, ML, JS, YW, and FC contributed to the writing and editing of the manuscript. All authors contributed to the article and approved the submitted version.

## Funding

The present study was supported by the Shanghai Yangpu District Health and Family Planning Commission Fund for Hao Yi Shi Training Project (Grant nos. 201742, 2020-2023) and the Natural Science Foundation of Shanghai (Grant no. 18ZR1436000).

## Conflict of Interest

Author YH was employed by company Cellomics International Limited.

The remaining authors declare that the research was conducted in the absence of any commercial or financial relationships that could be construed as a potential conflict of interest.
